# Integrative Analysis of MUC4 to Prognosis and Immune Infiltration in Pan-Cancer: Friend or Foe?

**DOI:** 10.3389/fcell.2021.695544

**Published:** 2021-07-16

**Authors:** Xiao-Peng Gao, Jie-Jie Dong, Tian Xie, Xiaoqing Guan

**Affiliations:** ^1^Department of Gastrointestinal Surgery, Yuncheng Central Hospital, Yuncheng, China; ^2^Department of Hepatopancreatobiliary Surgery, Yuncheng Central Hospital, Yuncheng, China; ^3^Department of Pediatrics, Yuncheng Central Hospital, Yuncheng, China; ^4^Key Laboratory of Carcinogenesis and Translational Research (Ministry of Education/Beijing), Center for Cancer Bioinformatics, Peking University Cancer Hospital and Institute, Beijing, China; ^5^Key Laboratory of Carcinogenesis and Translational Research (Ministry of Education/Beijing), Gastrointestinal Cancer Center, Peking University Cancer Hospital and Institute, Beijing, China

**Keywords:** mucin, prognosis, tumor immune microenvironment, pan-cancer, bioinformatic analysis

## Abstract

*MUC4*, a transmembrane mucin, plays important roles in epithelial renewal and differentiation. Recent studies suggest that *MUC4* has been implicated in pancreatic cancer pathogenesis and is expressed in various normal and cancer tissues. The underlying features of *MUC4* across various cancer types may allow us to ensure appropriate treatment and patient monitoring. However, the contributions of *MUC4* to pan-cancer have not been well characterized. In this study, we investigated the expression pattern and prognostic value of *MUC4* across multiple databases. We further explored genomic and epigenetic alterations of *MUC4*, its association with proliferation and metastasis, and the correlation with immune infiltration in different cancers. Our results characterized the distinct expression profile and prognostic values of *MUC4* in pan-cancer. Through examining its association with genomic alteration, tumor proliferation, and metastasis, as well as tumor infiltration, we revealed multiple function effects of *MUC4*. *MUC4* may influence prognosis, proliferation, metastasis, and immune response in opposite directions. In conclusion, our findings suggested the necessity to more carefully evaluate *MUC4* as a biomarker and therapeutic target and develop the new antibodies for cancer detection and intervention.

## Introduction

In general, mucins are a family of glycosylated proteins, which are expressed by epithelial cells and provide protection and lubrication to epithelial surfaces (Kufe, 2009). However, aberrant expression of mucins occurs in various cancers and has been implicated in cancer progression and prognosis (Bhatia et al., 2019).

MUC4, a transmembrane mucin, is localized on chromosome band 3q29. Human MUC4 was first identified in 1991 from a tracheal library (Carraway et al., 2009). MUC4 is synthesized as two subunits: MUC4α and MUC4β. MUC4α contains a tandem-repeat domain altering glycosylation and epitope multiplicity, a nidogen-like domain, and an adhesion-associated domain. MUC4β consists of a von Willebrand factor-type D domain and three epidermal growth factor (EGF)-like domains. On the basis of the specific structure, MUC4 was suggested to modulate HER2/ERBB2 signaling and play a critical role in cancer.

Normally, *MUC4* is expressed in the salivary glands, trachea and bronchioles, reproductive tract, colon, and mammary epithelium. In the past few years, many studies have reported that *MUC4* is aberrantly produced in a variety of cancers, including lung, breast, pancreatic, prostate, ovarian, and bladder, and functionally links to tumor initiation, metastasis, and interaction of tumor cells with the components of the tumor microenvironment. The available evidence indicates that *MUC4* is overexpressed in pancreatic cancer and contributes to the aggressiveness and metastasis of pancreatic cancer (Gautam et al., 2020; Sagar et al., 2021). *MUC4* is also overexpressed in ovarian cancer and promotes the pathobiology and aggressiveness of ovarian cancer cells (Ponnusamy et al., 2011; Bae et al., 2017). *MUC4* also plays a pivotal role in intestinal cell proliferation during tumorigenesis (Das et al., 2016). Rowson-Hodel et al. have provided considerable evidence that aberrantly expressed *MUC4* can lead to the metastatic efficiency of breast cancer (Rowson-Hodel et al., 2018). In contrast, *MUC4* expression was associated with improved survival and decreased recurrence in squamous cell carcinoma of the upper aerodigestive tract (Weed et al., 2004). These results suggested that the role of *MUC4* appears to be complicated depending on the particular cancer and cell context. Thus, the expression and function of *MUC4* in human tumors remain unclear and need to be analyzed in detail.

In our study, we conducted a comprehensive and profound bioinformatics analysis of *MUC4* expression and correlation with prognosis and immune infiltration in cancer patients through Oncomine (Rhodes et al., 2004), TIMER2.0 (Li et al., 2020), PrognoScan (Mizuno et al., 2009), Kaplan–Meier plotter (Nagy et al., 2021), GEPIA2 (Tang et al., 2019), UALCAN (Chandrashekar et al., 2017), TISIDB (Ru et al., 2019), cBioPortal (Gao et al., 2013), and CVCDAP (Guan et al., 2020). Our findings may elucidate its significant function in cancer pathogenesis and prospective uses in cancer diagnosis and prognosis and as a target for cancer immunotherapy.

## Materials and Methods

### Differential Expression Analysis

We compared mRNA expression levels of *MUC4* between normal and tumor tissues of each cancer type using Oncomine and GEPIA2. In Oncomine, a *t*-test was used to calculate the *p*-value, and the threshold was set as a *p*-value of 0.0001 and a fold change of 2. In GEPIA2, we used analysis of variance (ANOVA) for differential expression analysis and considered genes as differentially expressed genes with a fold change >2 and an FDR < 0.01. We also used GEPIA2 to assess the differential expression between different stages across independent cancer types by *t*-test and defined *p*-value <0.05 as significant.

### Survival Analysis

We performed overall survival analysis based on *MUC4* expression using PrognoScan and Kaplan–Meier Plotter. PrognoScan employed a univariate Cox regression model to find the optimal cut point in continuous gene expression measurement without prior biological knowledge or assumption and calculate the minimum *p*-value and hazard ratios with 95% confidence intervals for grouping patients. The Kaplan–Meier plotter split all patients into high- or low-expression groups according to the median value of *MUC4* and used the log-rank test for hypothesis testing. We also used cBioPortal to evaluate the prognosis of *MUC4* alteration. *p*-value <0.05 was considered significant.

### Characterize the Alteration and Methylation Profile in Pan-Cancer

We explored the genomic alteration frequency including non-synonymous mutation and copy number alteration of *MUC4* in the TCGA PanCancer Atlas dataset *via* cBioPortal. We obtained the promoter DNA methylation level of normal and tumor samples from UALCAN. Significance of difference was estimated by *t*-test. A *p*-value under 0.05 was considered statistically significant.

### Construct a PPI Network and Estimate the Associations Between MUC4 and Tumor Proliferation and Metastasis

We carried out PPI analysis with STRING, and the max number of interactions to show was 10. The proliferation marker ki67 was used to reflect tumor proliferation across tumor samples. We used CVCDAP to evaluate the correlation between *MUC4* and *MKI67* by Pearson’s correlation and considered |*R*| > 0.3 and *p*-value <0.05 to indicate significance. We also used CVCDAP to divide tumor samples into a high and low group by the median expression value of *MUC4* for each cancer type and calculate the epithelial–mesenchymal transition (EMT) enrichment score by GSEA. The gene set of EMT was collected from MSigDB. *FDR* < 0.25 was set as the threshold for screening.

### Correlation Between MUC4 and Immune Infiltration

We employed TIMER2.0 for correlation between *MUC4* and tumor immune infiltration. *Via* TIMER2.0, we analyzed the correlation between *MUC4* and six immune cells, including CD8^+^ and CD4^+^ T cells, B cells, macrophages, neutrophils, and dendritic cells, in KIRC and PAAD. We also explored the relationships between *MUC4* and immune gene markers in KIRC and PAAD. The association was generated with tumor purification adjusted. We used TISIDB to calculate the correlation between expression of *MUC4* and abundance of immunomodulators and chemokines. The correlation was statistically assessed by Spearman’s correlation. *p*-value <0.05 was considered significant.

## Results

### Differential Expression of MUC4

**FIGURE 1:**
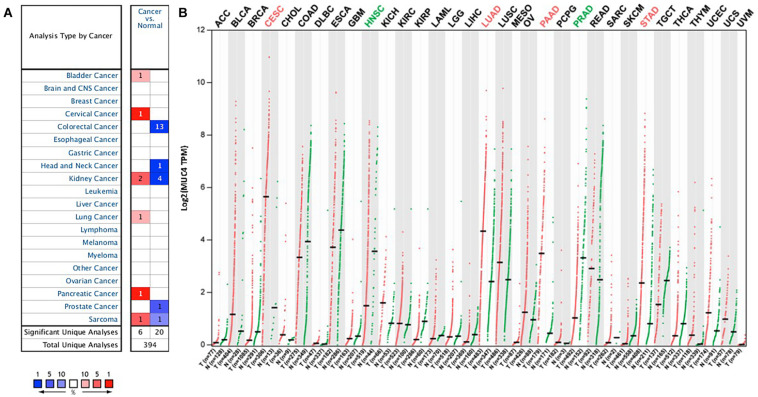
*MUC4* mRNA expression levels in pan-cancer. **(A)** Upregulated or downregulated expression of *MUC4* in tumor tissues, compared with normal tissues of each cancer type in Oncomine. Red signifies the gene’s overexpression in the analyses represented by that cell in the table; blue represents the gene’s underexpression in those analyses. Intensity of color signifies the best rank of that gene in those analyses. The number in each cell represents the number of analyses that meet your thresholds within those analyses and cancer types. **(B)** *MUC4* expression profile across all tumor samples and paired normal tissues in GEPIA2. Each dot represents a distinct tumor or normal sample.

To understand the differences in *MUC4* expression between human cancer and normal tissues, *MUC4* expression was explored *via* Oncomine. Our results revealed that *MUC4* expression is upregulated or downregulated in different types of cancer (Figure 1A). Compared to normal tissues, the expression of *MUC4* was significantly higher in bladder cancer, cervical cancer, lung cancer, and pancreatic cancer. In contrast, the expression of *MUC4* was lower in colorectal cancer, head and neck cancer, prostate cancer. Of interest, *MUC4* was upregulated in two datasets while it was downregulated in four datasets in kidney cancer. Similarly, *MUC4* was upregulated in one dataset while it was downregulated in one dataset in sarcoma.

We further confirmed the differential gene expression between tumor samples and normal tissues using TCGA datasets in GEPIA2 (Figure 1B). Compared to normal tissues, the expression of *MUC4* was significantly higher in CESC, LUAD, PAAD, and STAD, while the expression of *MUC4* was lower in HNSC and PRAD.

### Prognostic Analysis of MUC4

**FIGURE 2:**
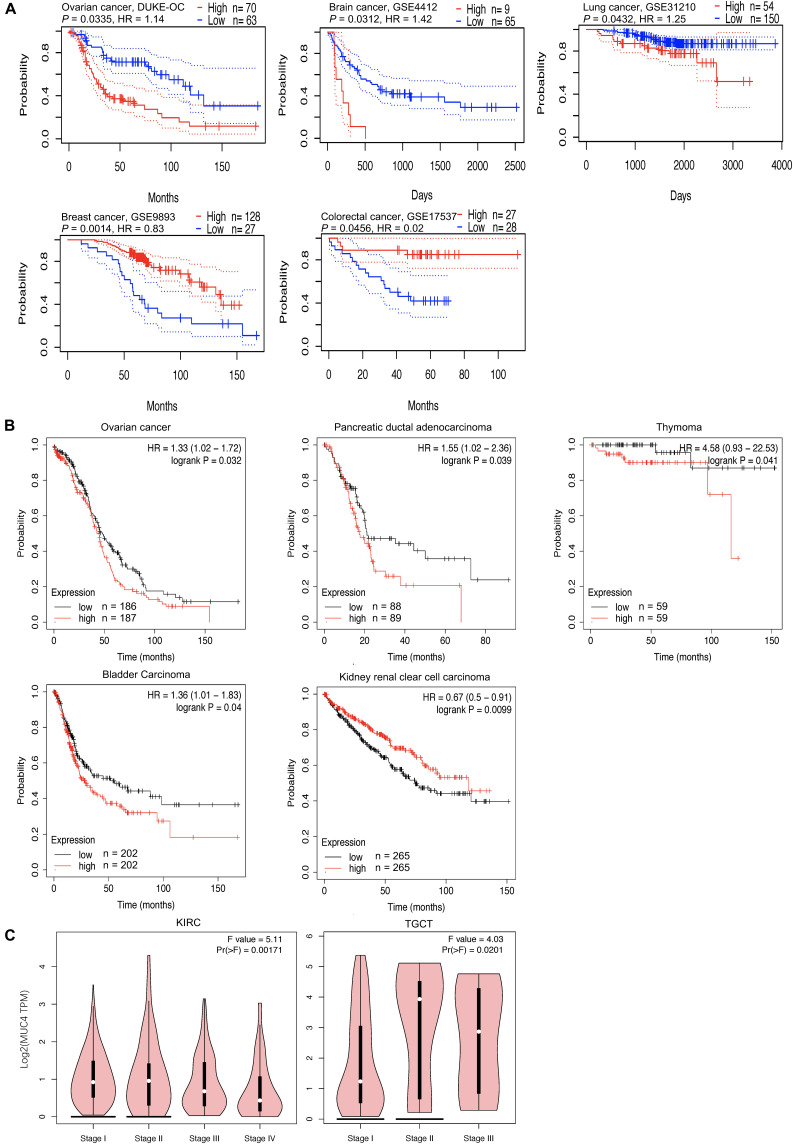
Overall survival curves comparing the high and low expression of *MUC4* and pathological stage plot across different cancer types. **(A,B)** Kaplan–Meier plot in PrognoScan panel **(A)** and Kaplan–Meier Plotter panel **(B)**. The X-axis represents time, and the Y-axis represents survival rate. Ninety-five percent confidence intervals for each group are also indicated by dotted lines from PrognoScan. **(C)** Expression violin plot based on patient pathological stage in GEPIA2. HR, hazard ratio.

In cancer research, the relevance of *MUC4* to clinical outcome may suggest the potential pathogenesis of disease and stimulate further researches. The impact of *MUC4* on overall survival was evaluated through PrognoScan (Figure 2A). The results showed that high expression of *MUC4* was associated with a poor prognosis in ovarian cancer (HR = 1.14, *p* = 0.0335), brain cancer (HR = 1.42, *p* = 0.0312), and lung cancer (HR = 1.25, *p* = 0.0335). However, in breast cancer (HR = 0.83, *p* = 0.0014) and colorectal cancer (HR = 0.02, *p* = 0.0456), increased expression of *MUC4* was significantly correlated with good survival.

In addition, we examined the potential effects of *MUC4* on prognosis across different cancer types *via* the Kaplan–Meier plotter (Figure 2B). Our study revealed that the poor prognosis of ovarian cancer (HR = 1.42, 95% CI: 1.02–1.72, *p* = 0.0312), pancreatic ductal adenocarcinoma (HR = 1.55, 95% CI: 1.02–2.36, *p* = 0.039), thymoma (HR = 4.58, 95% CI: 0.93–22.53, *p* = 0.041), and bladder cancer (HR = 1.36, 95% CI: 1.01–1.83, *p* = 0.04) was related to the high expression of *MUC4*. Furthermore, the increased expression of *MUC4* was related with prolonged overall survival in kidney renal clear cell carcinoma (HR = 0.67, 95% CI: 0.5–0.91, *p* = 0.0099). These findings revealed that the expression of *MUC4* has important significance in the prognosis in pan-cancer and can be used as a prognostic factor. To further understand the relevance of *MUC4* expression in cancer, we used the TCGA database to study the relationship between *MUC4* expression and pathological stage *via* GEPIA2 (Figure 2C). The *MUC4* expression profile observed in KIRC and TGCT may suggest a link between the level of *MUC4* and the tumor stage.

### Alteration Frequency and Methylation Level of MUC4

**FIGURE 3:**
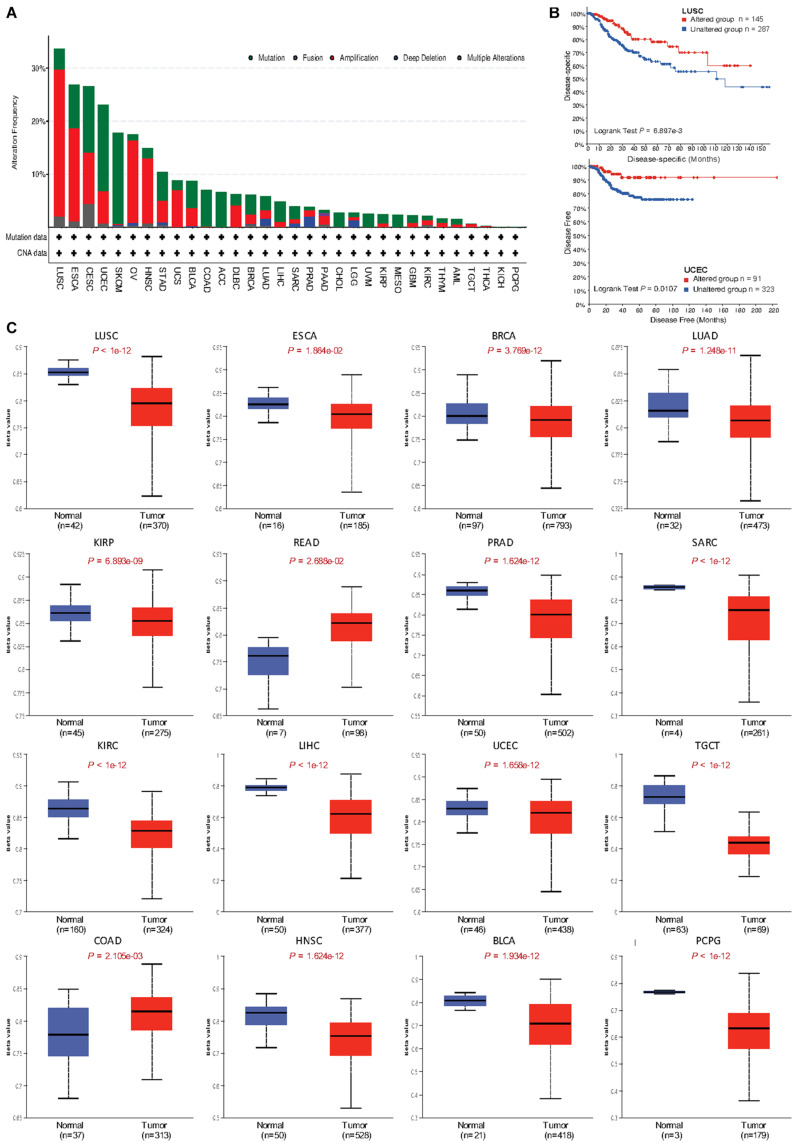
Genomic alteration and methylation of *MUC4* across different cancer types. **(A)** Alteration frequency of *MUC4* across different cancer types in cBioPortal. **(B)** The prognostic value of *MUC4* alteration in LUSC and UCEC. **(C)** *MUC4* promoter methylation level in tumor and normal samples of the TCGA database in UALCAN. The beta value indicates level of DNA methylation ranging from 0 (unmethylated) to 1 (fully methylated). Different beta value cutoffs have been considered to indicate hyper-methylation [beta value: 0.7–0.5] or hypo-methylation [beta-value: 0.3–0.25].

To identify the mechanism by which *MUC4* impacts survival, we used cBioPortal to explore alteration frequencies, including mutation, fusion, amplification, deep deletion, and multiple alterations, of *MUC4* in different cancer types (Figure 3A and Table 1). Results showed that the top five cancer types with more total mutations were LUSC (33.676%), ESCA (26.923%), CESC (26.599%), UCEC (23.062%), and SKCM (17.793%). For specific alteration types, amplifications of *MUC4* were enriched in LUSC (27.721%), ESAC (17.582%), OV (15.582%), HNSC (12.237%), and CESC (9.764%), while deep deletions were enriched in PRAD (2.024%), LGG (1.362%), LUAD (1.237%), SARC (0.784%), and OV 0.514%). SKCM (17.117%), UCEC (16.257%), CESC (12.458%), ESCA (8.242%), and COAD (6.902%) were the top five cancer types with more mutation frequencies. We also found one *MUC4-PCYT1A* fusion in PAAD (0.543%). Based on these results, we further studied the correlation of *MUC4* alteration with prognosis in top five cancer types and found the prognostic value of *MUC4* alteration. The results are summarized in Figure 3B. Altered *MUC4* is significantly associated with a good prognosis in LUSC (disease-specific survival, *p* = 6.8973-03) and UCEC (disease-free survival, *p* = 0.0107).

**TABLE 1:** Alteration frequency of *MUC4* across different cancer types in cBioPortal.

Cancer type	Cancer type details	Multiple alterations	Amplification	Deep deletion	Mutation	Fusion	Total
		(%)	(%)	(%)	(%)	(%)	(%)
LUSC	Lung squamous cell carcinoma	2.053	27.721		3.901		33.676
ESAC	Esophageal adenocarcinoma	1.099	17.582		8.242		26.923
CESC	Cervical squamous cell carcinoma	4.377	9.764		12.458		26.599
UCEC	Uterine corpus endometrial carcinoma	0.756	6.049		16.257		23.062
SKCM	Skin cutaneous melanoma	0.225	0.225	0.225	17.117		17.793
OV	Ovarian serous cystadenocarcinoma	0.342	15.582	0.514	1.027		17.466
HNSC	Head and neck squamous cell carcinoma	0.765	12.237		1.912		14.914
STAD	Stomach adenocarcinoma	0.455	4.091	0.455	5.455		10.455
UCS	Uterine carcinosarcoma		7.018		1.754		8.772
BLCA	Bladder urothelial carcinoma		3.406	0.243	5.109		8.759
COAD	Colorectal adenocarcinoma		0.168		6.902		7.071
ACC	Adrenocortical carcinoma				6.593		6.593
DLBC	Diffuse large B-cell lymphoma		4.167		2.083		6.250
BRCA	Breast invasive carcinoma	0.554	1.753	0.092	3.690		6.089
LUAD	Lung adenocarcinoma	0.353	1.590	1.237	2.650		5.830
LIHC	Liver hepatocellular carcinoma		1.075		3.763		4.839
SARC	Sarcoma		0.784	0.784	2.353		3.922
PRAD	Prostate adenocarcinoma		1.215	2.024	0.607		3.846
PAAD	Pancreatic adenocarcinoma	0.543	1.630		0.543	0.543	3.261
CHOL	Cholangiocarcinoma				2.778		2.778
LGG	Brain lower-grade glioma		0.584	1.362	0.778		2.724
UVM	Uveal melanoma				2.500		2.500
KIRP	Kidney renal papillary cell carcinoma		0.707		1.767		2.473
MESO	Mesothelioma				2.299		2.299
GBM	Glioblastoma multiforme		0.845		1.351		2.196
KIRC	Kidney renal clear cell carcinoma	0.391	0.978		0.783		2.153
THYM	Thymoma		0.813		0.813		1.626
AML	Acute myeloid leukemia		0.500		1.000		1.500
TGCT	Testicular germ cell tumors		0.671				0.671
THCA	Thyroid carcinoma		0.200				0.200
KICH	Kidney chromophobe						
PCPG	Pheochromocytoma and paraganglioma						

We wondered whether *MUC4* was differentially methylated between tumor and normal samples, and we used UALCAN to compare their methylation level in dependent cancer types. We found that methylation levels were different between normal and tumor tissues in 16 cancer types (Figure 3C). In LUSC (*p* < 1e-12), ESCA (*p* = 1.864e-02), BRCA (*p* = 3.769e-12), LUAD (*p* = 1.248e-11), KIRP (*p* = 6.893e-09), PRAD (*p* = 1.624e-12), SARC (*p* < 1e-12), KIRC (*p* < 1e-12), LIHC (*p* < 1e-12), UCEC (*p* = 1.658e-12), TGCT (*p* < 1e-12), HNSC (*p* = 1.624e-12), BLCA (*p* = 1.934e-12), and PCPG (*p* < 1e-12), *MUC4* was lowly methylated in tumor samples, while in READ (*p* = 2.688e-02) and COAD (*p* = 2.105e-03), *MUC4* was highly methylated in tumor samples.

### Functional Effects of MUC4 Associated With Proliferation and Metastasis

**FIGURE 4:**
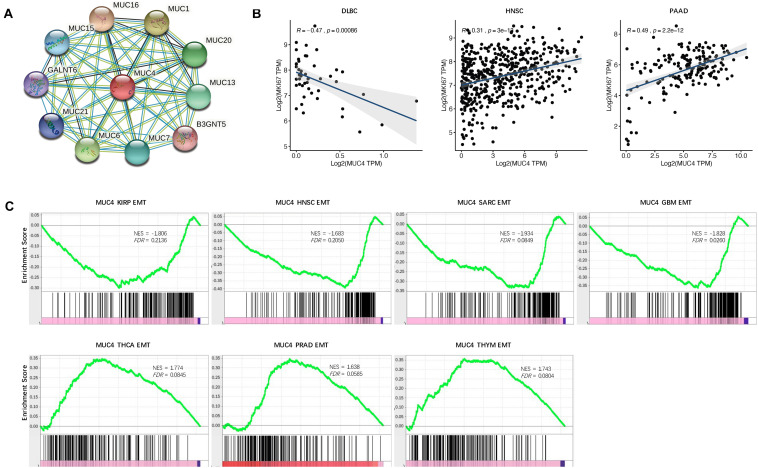
Functional effects of *MUC4*. **(A)** PPI network. Network nodes represent proteins and edges represent protein–protein associations, including both functional and physical protein associations. Line thickness indicates the strength of data support. **(B)** Correlation of *MUC4* with proliferation in CVCDAP. **(C)** Enrichment of *MUC4* in EMT in CVCDAP. Values of NES denote positive and negative enrichment. EMT, epithelial–mesenchymal transition.

To suspect the network of predicted associations for MUC4 and proteins with 10 best-scoring hits, we performed the PPI analysis and found that there were interactions between MUC4 and MUC16 (score = 0.986), MUC1 (score = 0.986), MUC6 (score = 0.984), MUC20 (score = 0.979), MUC13 (score = 0.979), MUC7 (score = 0.978), MUC15 (score = 0.971), MUC21 (score = 0.964), GRLANT6 (score = 0.957), and B3GNT5 (score = 0.947) (Figure 4A). All these proteins are critical in O-glycan processing, maintaining the gastrointestinal epithelium, and regulation of cell adhesion.

Cell proliferation is one of the significant hallmarks of cancer. To characterize the functional roles of *MUC4* in cell proliferation, we calculated the Rs between *MUC4* and the well-known proliferation marker *ki67* across cancer types. We identified a total of three significant associations (Figure 4B). *MUC4* negatively correlated with cell proliferation in DLBC (*R* = −0.47, *p* = 0.00086) and positively correlated with cell proliferation in HMSC (*R* = 0.31, *p* = 3e-13) and PAAD (*R* = 0.49, *p* = 2.2e-12).

Metastasis is the major cause of death among cancer patients. Recent studies have heralded that EMT plays a critical role in metastasis (Heerboth et al., 2015; Mittal, 2018). To investigate the functional roles of *MUC4* in metastasis, we assessed their enrichment associated with EMT through GSEA. We identified seven significant *MUC4* enrichments (*FDR* < 0.25) (Figure 4C). *MUC4* was negatively enriched in four cancer types, including KIRP (NES = −1.806, *FDR* = 0.2136), HNSC (NES = −1.683, *FDR* = 0.205), SARC (NES = −1.934, *FDR* = 0.0849), and GBM (NES = −1.828, *FDR* = 0.026). In contrast, *MUC4* was positively enriched in three cancer types, including THCA (NES = 1.774, *FDR* = 0.0845), PRAD (NES = 1.638, *FDR* = 0.0585), and THYM (NES = 1.743, *FDR* = 0.0804).

### Correlation Between MUC4 and Immune Infiltration Level

**FIGURE 5:**
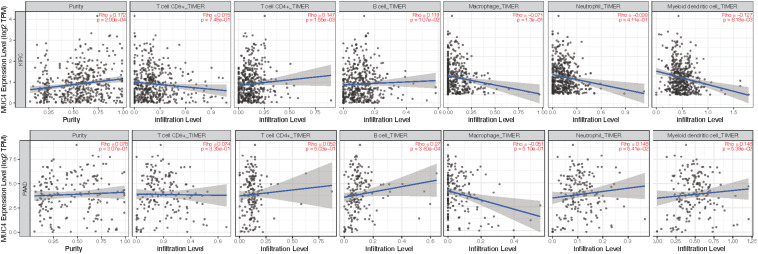
Coefficients for correlation between *MUC4* and immune cell infiltration of KIRC and PAAD in TIMER2.0.

Given the distinctive roles of *MUC4* in immunomodulation during cancer progression and metastasis (Yang et al., 2020; Peng et al., 2021), we used TIMER2.0 to investigate the impact of the expression of *MUC4* on tumor immune infiltration levels. The detailed results are shown in Figure 5. According to the TIMER2.0 results, we identified *MUC4* expression has weak positive relevance with tumor purity and the immune-infiltrating levels of CD4^+^ T cells (*R* = 0.147, *p* = 1.55e-03) and B cells (*R* = 0.119, *p* = 1.07e-02) but a negative correlation with dendritic cells (*R* = −0.127, *p* = 6.19e-03) in KIRC. In PAAD, *MUC4* expression has significant positive correlations with the immune-infiltrating levels of B cells (*R* = 0.27, *p* = 3.6e-04) but no significant correlation with the infiltrating levels of CD8^+^ T cells, CD4^+^ T cells, macrophages, neutrophils, and dendritic cells.

**FIGURE 6:**
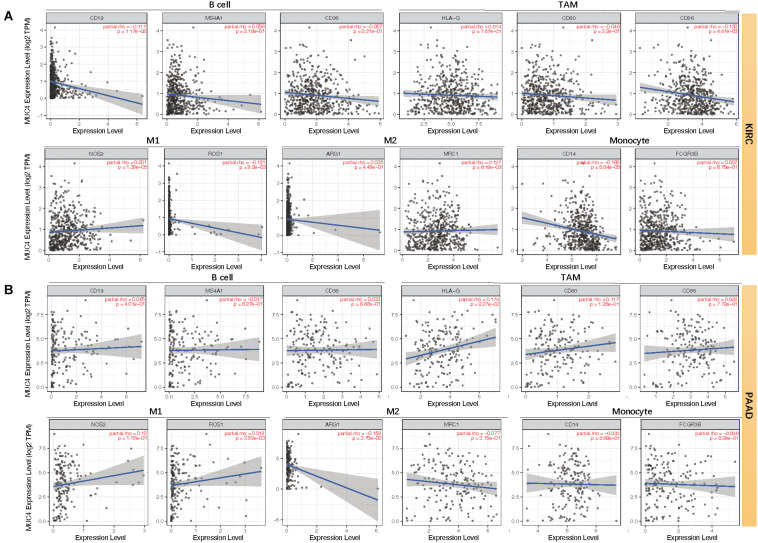
Correlation between mRNA expression of *MUC4* and immune markers of KIRC **(A)** and PAAD **(B)** in TIMER2.0. TAM, tumor-associated macrophages; M1, type-1 macrophage; M2, type-2 macrophage.

**FIGURE 7:**
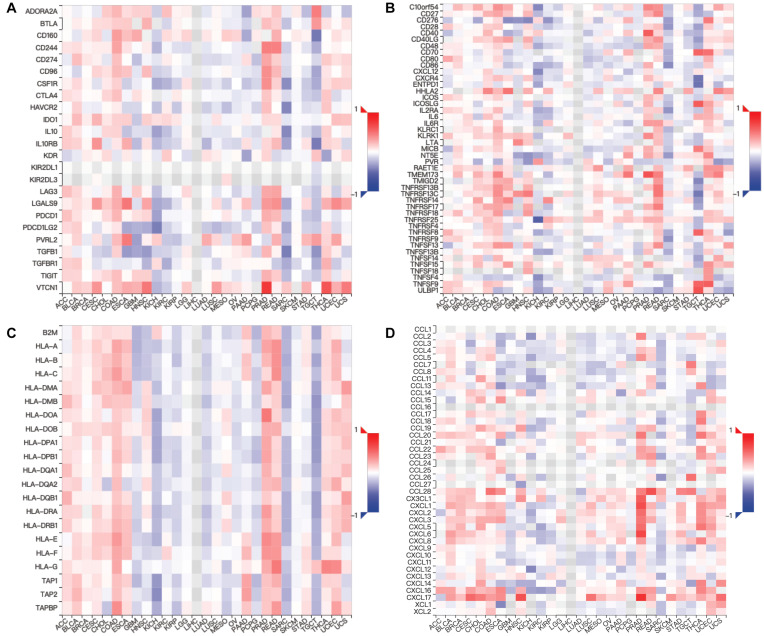
Relations between expression of *MUC4* and three kinds of immunomodulators and chemokines in TISIDB. Spearman correlations between expression of *MUC4* and immunoinhibitors **(A)**, immunostimulators **(B)**, MHCs **(C)**, and chemokines **(D)** across human cancers.

Moreover, we assessed the relevance between *MUC4* and immune marker genes to clarify the mechanism of *MUC4* in immune regulation in cancers (Figure 6). After adjusting the correlations by purity, we found that *MUC4* expression has a positive correlation with *NOS2* (M1) (*R* = 0.201, *p* = 1.39e-05) and *MRC1* (M2) (*R* = 0.127, *p* = 6.18e-03) but negative relevance to *CD19* (B cell) (*R* = −0.117, *p* = 1.17e-02), *CD86* (tumor-associated macrophages, TAM) (*R* = −0.132, *p* = 4.41e-03), *ROS1* (M1) (*R* = −0.121, *p* = 9.2e-03), and *CD14* (monocyte) (*R* = −0.188, *p* = 5.04e-05) in KIRC. *MUC4* expression in PAAD has a weak to moderate positive correlation with the expression of gene marker sets of TAM (*HLA-G*) (*R* = 0.174, *p* = 2.27e-02) and M1 (*ROS1*) (*R* = 0.219, *p* = 3.92e-03) but a negative correlation with M2 (*ARG1*) (*R* = −0.159, *p* = 3.78e-02). We also found that the relevance of *MUC4* expression to other gene marker sets was not significant, such as B cell (*CD19*, *MS4A1*, *CD38*), TAM (*CD80* and *CD86*), M1 (*NOS2*), M2 (*MRC1*), and monocyte (*CD14* and *FCGR3B*). We further studied the correlations between *MUC4* and three kinds of immunomodulators and chemokines in UALCAN. The detailed results are described in Figure 7.

## Discussion

Past research has shown the critical roles of mucins in tumorigenesis (van Putten and Strijbis, 2017; Maeda et al., 2018; Ganguly et al., 2020; Liu et al., 2020), which indicated their prospective usefulness in cancer diagnosis, prognosis, and therapy (Nabavinia et al., 2017; Aithal et al., 2018; Guo et al., 2018; Lucchetta et al., 2019; Pothuraju et al., 2020). Previous studies have raised hope that *MUC4* can be a good candidate marker for pancreatic malignancy (Trabbic et al., 2019; Gautam et al., 2020). *MUC4* could also be used in combination with *MUC16* for detection of advanced ovarian cancer (Chauhan et al., 2006). However, it remains unclear whether *MUC4* can be characterized as a friend or foe across the cancer types (Jonckheere and Van Seuningen, 2018). To address this issue, we performed an integrative analysis about *MUC4* to understand its effect on survival and immunomodulation, which is necessary to develop the *MUC4*-based cancer therapy. Through a comprehensive analysis in large-scale datasets, we identified consistent expression levels of *MUC4* in pan-cancer using independent datasets *via* Oncomine and GEPIA2, which showed that *MUC4* expression compared with normal samples was upregulated in cervical cancer, lung cancer, and pancreatic cancer, while it was downregulated in head and neck cancer and prostate cancer. We analyzed its prognostic significance statistically *via* PrognoScan and Kaplan–Meier plotter. Increased *MUC4* expression was significantly correlated with prolonged survival time in breast cancer, colorectal cancer, and kidney renal cell carcinoma, while it was associated with poor survival in ovarian cancer, brain cancer, lung cancer, pancreatic cancer, thymoma, bladder carcinoma. Overall, these findings strongly suggest that *MUC4* can serve as a prognostic biomarker in pan-cancer.

Furthermore, we explored the mechanism by which *MUC4* influences prognosis. We depicted global alterations and epigenetic regulation of *MUC4* across multiple cancer types, which showed that genomic alteration was an unfavorable factor for overall survival in LUSC and UCEC. As we expected, alteration in mRNA level and genomic level may influence tumor-malignant traits through different mechanisms. A previous study showed a high correlation between hypomethylation status and mRNA expression *MUC4*, and patients with *MUC4* hypomethylation correlated with poor prognosis in pancreatic cancer (Yokoyama et al., 2016). Yamada et al. reported that the mRNA expression of *MUC4* negatively correlated with its DNA methylation status at promoter regions in human lung cancer cell lines (Yamada et al., 2009), which was consistent with our results.

The effects on protein network, tumor growth, and metastasis were also concerned. Highly expressed *MUC4* was correlated with *MKI67* expression and negatively enriched with EMT in HNSC, which was consistent with its expression profile. In HNSC, upregulated *MUC4* expression was enriched with EMT, which was identical with its poor prognosis. In addition, we focused on the functional roles on the tumor-associated microenvironment, especially in KIRC and PAAD. Our results demonstrated that the correlation with immune gene markers was not always the same as the overall trend (the relationships with immune cells). For example, in PAAD, B cells significantly correlated with *MUC4* expression; however, gene markers of B cell had no relation to *MUC4* expression. These discrepant implications on immune response and survival need further experiments for validation.

When considered together, our data demonstrated that *MUC4* expression and promoter methylation status are potential prognosis biomarkers for lung cancer. *MUC4* can be explored in pancreatic cancer as an early diagnostic tool. Thus, these findings in our study may provide new avenues for understanding the biological characterization of *MUC4* and make sense in the design of therapeutic strategies.

## Data Availability Statement

The original contributions presented in the study are included in the article/supplementary material, further inquiries can be directed to the corresponding author.

## Author Contributions

X-PG and XG led the bioinformatic and biostatistical data analysis. X-PG and J-JD collected the literature, wrote the manuscript, and made the figures. XG and TX edited and made significant revisions to the manuscript. XG contributed to the study design and project supervision. All authors contributed to the article and approved the submitted version.

## Conflict of Interest

The authors declare that the research was conducted in the absence of any commercial or financial relationships that could be construed as a potential conflict of interest.

## References

[B1] Aithal A. Rauth S. Kshirsagar P. Shah A. Lakshmanan I. Junker W. M. (2018). MUC16 as a novel target for cancer therapy. *Expert Opin. Ther. Targets* 22 675–686. 29999426 10.1080/14728222.2018.1498845 PMC6300140

[B2] Bae J. S. Lee J. Park Y. Park K. Kim J. R. Cho D. H. (2017). Attenuation of MUC4 potentiates the anticancer activity of auranofin via regulation of the Her2/Akt/FOXO3 pathway in ovarian cancer cells. *Oncol. Rep.* 38 2417–2425. 10.3892/or.2017.5853 28765909

[B3] Bhatia R. Gautam S. K. Cannon A. Thompson C. Hall B. R. Aithal A. (2019). Cancer-associated mucins: role in immune modulation and metastasis. *Cancer Metastasis Rev.* 38 223–236. 10.1007/s10555-018-09775-0 30618016 PMC6614013

[B4] Carraway K. L. Theodoropoulos G. Kozloski G. A. Carothers Carraway C. A. (2009). Muc4/MUC4 functions and regulation in cancer. *Future Oncol.* 5 1631–1640. 10.2217/fon.09.125 20001800 PMC2825673

[B5] Chandrashekar D. S. Bashel B. Balasubramanya S. A. H. Creighton C. J. Ponce-Rodriguez I. Chakravarthi B. (2017). UALCAN: a portal for facilitating tumor subgroup gene expression and survival analyses. *Neoplasia* 19 649–658. 10.1016/j.neo.2017.05.002 28732212 PMC5516091

[B6] Chauhan S. C. Singh A. P. Ruiz F. Johansson S. L. Jain M. Smith L. M. (2006). Aberrant expression of MUC4 in ovarian carcinoma: diagnostic significance alone and in combination with MUC1 and MUC16 (CA125). *Mod. Pathol.* 19 1386–1394. 10.1038/modpathol.3800646 16880776

[B7] Das S. Rachagani S. Sheinin Y. Smith L. M. Gurumurthy C. B. Roy H. K. (2016). Mice deficient in Muc4 are resistant to experimental colitis and colitis-associated colorectal cancer. *Oncogene* 35 2645–2654. 10.1038/onc.2015.327 26364605 PMC5555307

[B8] Ganguly K. Rauth S. Marimuthu S. Kumar S. Batra S. K. (2020). Unraveling mucin domains in cancer and metastasis: when protectors become predators. *Cancer Metastasis Rev.* 39 647–659. 10.1007/s10555-020-09896-5 32488403 PMC7487023

[B9] Gao J. Aksoy B. A. Dogrusoz U. Dresdner G. Gross B. Sumer S. O. (2013). Integrative analysis of complex cancer genomics and clinical profiles using the cBioPortal. *Sci. Signal.* 6:l1. 10.1126/scisignal.2004088 PMC4160307 23550210

[B10] Gautam S. K. Kumar S. Dam V. Ghersi D. Jain M. Batra S. K. (2020). MUCIN-4 (MUC4) is a novel tumor antigen in pancreatic cancer immunotherapy. *Semin. Immunol.* 47:101391. 10.1016/j.smim.2020.101391 31952903 PMC7160012

[B11] Guan X. Cai M. Du Y. Yang E. Ji J. Wu J. (2020). CVCDAP: an integrated platform for molecular and clinical analysis of cancer virtual cohorts. *Nucleic Acids Res.* 48 W463–W471. 32449936 10.1093/nar/gkaa423 PMC7439093

[B12] Guo M. You C. Dou J. (2018). Role of transmembrane glycoprotein mucin 1 (MUC1) in various types of colorectal cancer and therapies: current research status and updates. *Biomed. Pharmacother.* 107 1318–1325. 10.1016/j.biopha.2018.08.109 30257347

[B13] Heerboth S. Housman G. Leary M. Longacre M. Byler S. Lapinska K. (2015). EMT and tumor metastasis. *Clin. Transl. Med.* 4:6. 10.1186/s40169-015-0048-3 PMC4385028 25852822

[B14] Jonckheere N. Van Seuningen I. (2018). Integrative analysis of the cancer genome atlas and cancer cell lines encyclopedia large-scale genomic databases: MUC4/MUC16/MUC20 signature is associated with poor survival in human carcinomas. *J. Transl. Med.* 16:259. 10.1186/s12967-018-1632-2 PMC6149062 30236127

[B15] Kufe D. W. (2009). Mucins in cancer: function, prognosis and therapy. *Nat. Rev. Cancer* 9 874–885. 10.1038/nrc2761 19935676 PMC2951677

[B16] Li T. Fu J. Zeng Z. Cohen D. Li J. Chen Q. (2020). TIMER2.0 for analysis of tumor-infiltrating immune cells. *Nucleic Acids Res.* 48 W509–W514. 32442275 10.1093/nar/gkaa407 PMC7319575

[B17] Liu F. Fu J. Bergstrom K. Shan X. McDaniel J. M. McGee S. (2020). Core 1-derived mucin-type O-glycosylation protects against spontaneous gastritis and gastric cancer. *J. Exp. Med.* 217:e20182325. 10.1084/jem.20182325 31645367 PMC7037257

[B18] Lucchetta M. da Piedade I. Mounir M. Vabistsevits M. Terkelsen T. Papaleo E. (2019). Distinct signatures of lung cancer types: aberrant mucin O-glycosylation and compromised immune response. *BMC Cancer* 19:824. 10.1186/s12885-019-5965-x 31429720 PMC6702745

[B19] Maeda T. Hiraki M. Jin C. Rajabi H. Tagde A. Alam M. (2018). MUC1-C induces PD-L1 and immune evasion in triple-negative breast cancer. *Cancer Res.* 78 205–215. 10.1158/0008-5472.can-17-1636 29263152 PMC5754244

[B20] Mittal V. (2018). Epithelial mesenchymal transition in tumor metastasis. *Annu. Rev. Pathol.* 13 395–412. 29414248 10.1146/annurev-pathol-020117-043854

[B21] Mizuno H. Kitada K. Nakai K. Sarai A. (2009). PrognoScan: a new database for meta-analysis of the prognostic value of genes. *BMC Med. Genomics* 2:18. 10.1186/1755-8794-2-18 19393097 PMC2689870

[B22] Nabavinia M. S. Gholoobi A. Charbgoo F. Nabavinia M. Ramezani M. Abnous K. (2017). Anti-MUC1 aptamer: a potential opportunity for cancer treatment. *Med. Res. Rev.* 37 1518–1539. 10.1002/med.21462 28759115

[B23] Nagy A. Munkacsy G. Gyorffy B. (2021). Pancancer survival analysis of cancer hallmark genes. *Sci. Rep.* 11:6047. 10.1038/s41598-021-84787-5 PMC7961001 33723286

[B24] Peng L. Li Y. Gu H. Xiang L. Xiong Y. Wang R. (2021). Mucin 4 mutation is associated with tumor mutation burden and promotes antitumor immunity in colon cancer patients. *Aging* 13 9043–9055. 10.18632/aging.202756 33714943 PMC8034916

[B25] Ponnusamy M. P. Seshacharyulu P. Vaz A. Dey P. Batra S. K. (2011). MUC4 stabilizes HER2 expression and maintains the cancer stem cell population in ovarian cancer cells. *J. Ovarian Res.* 4:7. 10.1186/1757-2215-4-7 21521521 PMC3111401

[B26] Pothuraju R. Rachagani S. Krishn S. R. Chaudhary S. Nimmakayala R. K. Siddiqui J. A. (2020). Molecular implications of MUC5AC-CD44 axis in colorectal cancer progression and chemoresistance. *Mol. Cancer* 19:37. 10.1186/s12943-020-01156-y PMC7041280 32098629

[B27] Rhodes D. R. Yu J. Shanker K. Deshpande N. Varambally R. Ghosh D. (2004). ONCOMINE: a cancer microarray database and integrated data-mining platform. *Neoplasia* 6 1–6. 10.1016/s1476-5586(04)80047-2 15068665 PMC1635162

[B28] Rowson-Hodel A. R. Wald J. H. Hatakeyama J. O’Neal W. K. Stonebraker J. R. VanderVorst K. (2018). Membrane mucin Muc4 promotes blood cell association with tumor cells and mediates efficient metastasis in a mouse model of breast cancer. *Oncogene* 37 197–207. 10.1038/onc.2017.327 28892049 PMC5930013

[B29] Ru B. Wong C. N. Tong Y. Zhong J. Y. Zhong S. S. W. Wu W. C. (2019). TISIDB: an integrated repository portal for tumor-immune system interactions. *Bioinformatics* 35 4200–4202. 10.1093/bioinformatics/btz210 30903160

[B30] Sagar S. Leiphrakpam P. D. Thomas D. McAndrews K. L. Caffrey T. C. Swanson B. J. (2021). MUC4 enhances gemcitabine resistance and malignant behaviour in pancreatic cancer cells expressing cancer-associated short O-glycans. *Cancer Lett.* 503 91–102. 10.1016/j.canlet.2021.01.015 33485947 PMC7981252

[B31] Tang Z. Kang B. Li C. Chen T. Zhang Z. (2019). GEPIA2: an enhanced web server for large-scale expression profiling and interactive analysis. *Nucleic Acids Res.* 47 W556–W560. 31114875 10.1093/nar/gkz430 PMC6602440

[B32] Trabbic K. R. Whalen K. Abarca-Heideman K. Xia L. Temme J. S. Edmondson E. F. (2019). A tumor-selective monoclonal antibody from immunization with a tumor-associated mucin glycopeptide. *Sci. Rep.* 9:5662. 10.1038/s41598-019-42076-2 PMC6450958 30952968

[B33] van Putten J. P. M. Strijbis K. (2017). Transmembrane mucins: signaling receptors at the intersection of inflammation and cancer. *J. Innate Immun.* 9 281–299. 10.1159/000453594 28052300 PMC5516414

[B34] Weed D. T. Gomez-Fernandez C. Yasin M. Hamilton-Nelson K. Rodriguez M. Zhang J. (2004). MUC4 and ErbB2 expression in squamous cell carcinoma of the upper aerodigestive tract: correlation with clinical outcomes. *Laryngoscope* 114(8 Pt 2 Suppl. 101) 1–32. 10.1097/00005537-200408001-00001 15284539

[B35] Yamada N. Nishida Y. Tsutsumida H. Goto M. Higashi M. Nomoto M. (2009). Promoter CpG methylation in cancer cells contributes to the regulation of MUC4. *Br. J. Cancer* 100 344–351. 10.1038/sj.bjc.6604845 19127263 PMC2634723

[B36] Yang Y. Zhang J. Chen Y. Xu R. Zhao Q. Guo W. (2020). MUC4, MUC16, and TTN genes mutation correlated with prognosis, and predicted tumor mutation burden and immunotherapy efficacy in gastric cancer and pan-cancer. *Clin. Transl. Med.* 10:e155. 10.1002/ctm2.155 PMC7443139 32898332

[B37] Yokoyama S. Higashi M. Kitamoto S. Oeldorf M. Knippschild U. Kornmann M. (2016). Aberrant methylation of MUC1 and MUC4 promoters are potential prognostic biomarkers for pancreatic ductal adenocarcinomas. *Oncotarget* 7 42553–42565. 10.18632/oncotarget.9924 27283771 PMC5173155

